# Heart Rate Measurements in Patients with Obstructive Sleep Apnea and Atrial Fibrillation: Prospective Pilot Study Assessing Apple Watch’s Agreement With Telemetry Data

**DOI:** 10.2196/18050

**Published:** 2021-02-08

**Authors:** Pauline Huynh, Rongzi Shan, Ngozi Osuji, Jie Ding, Nino Isakadze, Francoise A Marvel, Garima Sharma, Seth S Martin

**Affiliations:** 1 School of Medicine Johns Hopkins University Baltimore, MD United States; 2 Ciccarone Center for the Prevention of Cardiovascular Disease Division of Cardiology, Department of Medicine Johns Hopkins University School of Medicine Baltimore, MD United States; 3 David Geffen School of Medicine University of California Los Angeles, CA United States

**Keywords:** mHealth, wearables, atrial fibrillation, obstructive sleep apnea, digital health

## Abstract

**Background:**

Patients with obstructive sleep apnea (OSA) are at a higher risk for atrial fibrillation (AF). Consumer wearable heart rate (HR) sensors may be a means for passive HR monitoring in patients with AF.

**Objective:**

The aim of this study was to assess the Apple Watch’s agreement with telemetry in measuring HR in patients with OSA in AF.

**Methods:**

Patients with OSA in AF were prospectively recruited prior to cardioversion/ablation procedures. HR was sampled every 10 seconds for 60 seconds using telemetry and an Apple Watch concomitantly. Agreement of Apple Watch with telemetry, which is the current gold-standard device for measuring HR, was assessed using mixed effects limits agreement and Lin’s concordance correlation coefficient.

**Results:**

A total of 20 patients (mean 66 [SD 6.5] years, 85% [n=17] male) participated in this study, yielding 134 HR observations per device. Modified Bland–Altman plot revealed that the variability of the paired difference of the Apple Watch compared with telemetry increased as the magnitude of HR measurements increased. The Apple Watch produced regression-based 95% limits of agreement of 27.8 – 0.3 × average HR – 15.0 to 27.8 – 0.3 × average HR + 15.0 beats per minute (bpm) with a mean bias of 27.8 – 0.33 × average HR bpm. Lin’s concordance correlation coefficient was 0.88 (95% CI 0.85-0.91), suggesting acceptable agreement between the Apple Watch and telemetry.

**Conclusions:**

In patients with OSA in AF, the Apple Watch provided acceptable agreement with HR measurements by telemetry. Further studies with larger sample populations and wider range of HR are needed to confirm these findings.

## Introduction

Atrial fibrillation (AF) is the most common clinically significant cardiac arrhythmia, with a lifetime risk of 1 in 4 among individuals over the age of 40 and about 1 in 3 among individuals over the age of 55, thereby posing substantial public health and economic burden [1,2]. AF is associated with significant cardiovascular and cerebrovascular morbidity and mortality, including a fivefold risk of thromboembolic complications such as stroke [1,3]. Because of its episodic, paroxysmal, and minimally symptomatic nature, the diagnosis of AF is often delayed, with nearly 1 in 5 diagnoses occurring at the onset of acute stroke [4]. It is estimated that nearly 700,000 people in the United States alone have undiagnosed AF due to its “clinically silent” nature, presenting a diagnostic challenge for clinicians [5].

Of particularly high risk for developing AF are individuals with sleep breathing disorders, including obstructive sleep apnea (OSA). A strong association between OSA and AF has been consistently observed in both epidemiological and clinical cohorts, with patients with OSA being 2 to 4 times more likely to develop AF compared to those without OSA [6-8]. Gami et al [9] reported significantly higher prevalence (49% vs 32%) of OSA and a strong association (adjusted odds ratio of 2.19) between OSA and AF in patients undergoing electrical cardioversion as compared to patients without AF. Moreover, a comorbid diagnosis of OSA is predictive of AF recurrences after catheter ablation or electrical cardioversion of AF [7,10].

Recently, the growing prevalence and adoption of digital health tools, including mobile devices with physiologic sensors (eg, “wearables”), have caught the attention of industry giants in the technology sector and clinicians who see opportunities for synergy in subclinical AF detection. This is evidenced by the rapid development and release of wearables for AF detection, including the Apple Watch Series 4 (Apple Inc.), KardiaBand and KardiaMobile (AliveCor), Hexoskin (Carré Technologies Inc.), and QardioCore (Qardio Inc.) [11]. Of these, only the Apple Watch Series 4 and KardiaMobile have been FDA cleared for AF detection [12,13], although many still list claims promoting heart health and wellness. Furthermore, ownership of wearables has more than doubled between 2014 and 2018 (from 25.1 million to 51.9 million users), and is further projected to increase with nearly half of the American public showing interest in future ownership [11,14].

Many wearables monitor heart rate (HR) through an optic technology known as photoplethysmography (PPG), in which sensors detect and measure pulsatile light absorption in the vasculature beneath the skin as a proxy for the cardiac cycle [15]. While this intersection in health technology has spurred numerous validation studies in the detection of AF [3,14,16], little is known about the accuracy of PPG technology in measuring HR during AF. Preliminary work by a single group in Australia suggests that during AF episodes, smart watches underestimate HR over 100 beats per minute (bpm) when compared to electrocardiogram (ECG) or Holter monitoring [17,18]. Similarly, as wearables evolve to accurately detect AF and bring users into the health care system, little research exists on how these technologies may also be used to help patients assess their AF management plans, which may include a rate control strategy and detection of rapid ventricular response (RVR).

In this pilot study, we assessed the Apple Watch’s agreement with telemetry as the gold standard in measuring HR in patients with OSA in AF. We chose to recruit patients with OSA given their higher likelihood of having a co-diagnosis of AF [19] and because we had encountered in clinical practice patients with OSA who had self-identified AF with RVR by a fast HR on their Apple Watch. We hypothesized that the Apple Watch would measure HR accurately when compared to standard ECG monitoring in patients with OSA in AF.

## Methods

### Study Approval

This study was approved by the Johns Hopkins Medicine Institutional Review Board. Apple Inc. was not involved in the design, implementation, data analysis, or manuscript preparation of the study.

### Study Design

In this prospective pilot study, patients aged 18 and older with OSA in AF episodes confirmed on ECG were identified via electronic health record screening and prospectively recruited prior to cardioversion and AF ablation procedures at Johns Hopkins Hospital between November 2018 and May 2019. Diagnosis of OSA was determined by chart review, and patients with objective clinical documentation of (1) current continuous positive airway pressure (CPAP) device use, (2) polysomnogram results showing OSA, or (3) both were considered eligible. Patients were excluded if they had implantable pacemakers, defibrillators, loop recorders, heart block, or tachycardia not attributable to AF. In addition, patients who were hemodynamically unstable or under contact precautions for infection control were excluded.

### Data Collection

**Figure 1:**
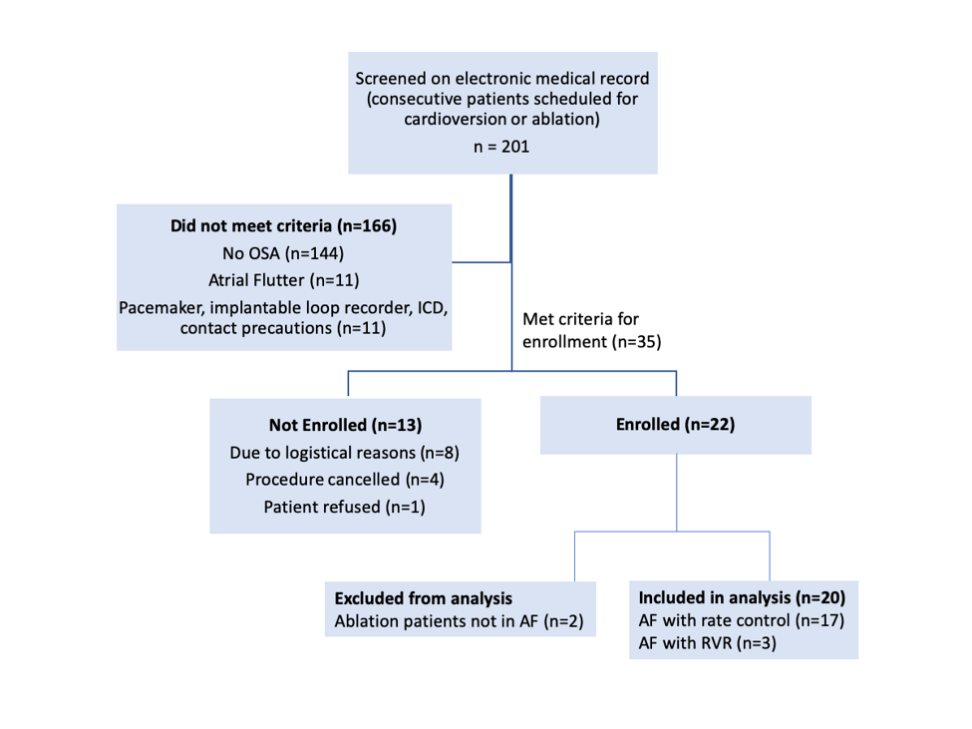
Study enrollment flowchart. ICD: implantable cardioverter-defibrillator. OSA: obstructive sleep apnea. AF: atrial fibrillation. RVR: rapid ventricular response.

Eligible patients were approached prior to their procedures and provided informed written consent. AF was confirmed by a 12-lead ECG performed minutes prior to HR data collection. Participants wore a first-generation Apple Watch (model A1554), which was provided by the study team for the duration of data collection. The same device was used for all participants and was cleaned between use with a hospital-grade disinfectant. The Apple Watch face and telemetry monitor (CARESCAPE Monitor B650; GE Healthcare) were observed concomitantly under video recording in the presence of a study co-investigator (RS) for 90 seconds. After excluding the first 30 seconds of data to allow time for the watch’s HR monitor to equilibrate, HR measurements were sampled every 10 seconds for 60 seconds, yielding a total of 7 observations per participant per device (Apple Watch and telemetry). In addition, we documented the following relevant clinical data: cardiac history, cardiovascular medications, OSA treatment, nature of AF diagnosis, and demographic characteristics using the electronic health record. Full study flow can be found in Figure 1.

### Statistical Analysis

**Figure 2:**
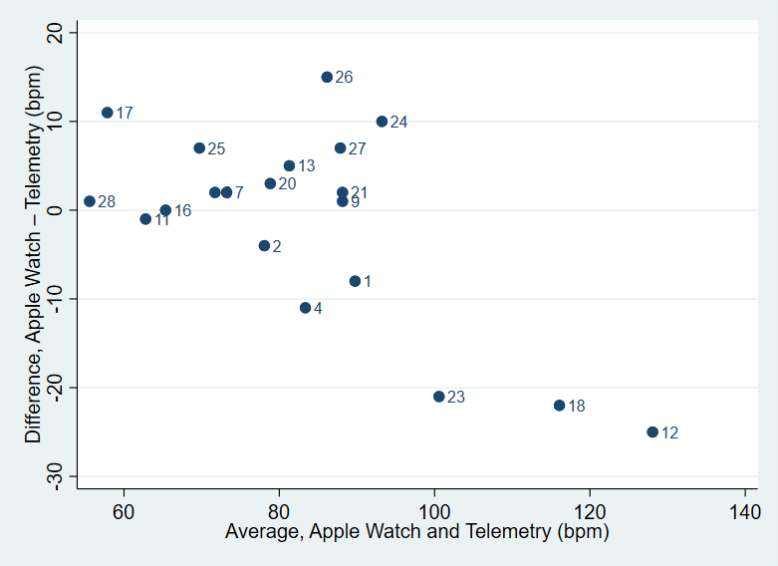
Scatter plots of standard deviation of measurement pair differences against patient mean. (Patients 23, 18, 12 had rapid ventricular response.) Here, we show a relationship between difference and magnitude of measurement, suggesting a violation of constant bias assumption.

Descriptive statistics were performed for the baseline characteristics, using frequencies (percentages) to describe categorical variables and mean (SD) or median (interquartile range) to describe continuous variables. Using the telemetry-determined HR as the gold standard, the Apple Watch was assessed for accuracy by calculating the paired difference between the measures. We first checked the mean constant bias assumption by visualizing the modified Bland–Altman plot accounting for repeated measures per patient (Figure 2). The mean bias appeared to be greater for higher HR measurements than for lower ones and log transformation of the data did not remove such relationship. We then analyzed the paired differences of Apple Watch compared with telemetry using a mixed effects regression model, with patients as a random effect and the averaged HR as the fixed effect. The paired difference was modeled in the following form [17]:

Diff_ijk_ = α + r_i_ + β_k_A + e_ij_

ri ~ N(0, δ_r_^2^), e_ij_ ~ N(0, δ_e_^2^)

where *Diff_ijk_* represents the *j*th paired difference in HR between devices in patient *i* given *k* value of the true (average) measurement; *α* is the constant intercept; *r_i_* is the random effect of the *i*th patient; *β_k_* is the fixed effect of average of 2 measurements: and *e_ij_* is the error for paired difference *j* on patient *i*.^17^ The regression of *Diff_ijk_* on the fixed effect of average of measurements gave the following:

*Diff_ijk_* = 27.7922 – 0.3332*A*

The coefficient of –0.3332 was statistically significant (*P*<.05) and further confirmed the average difference was related to the magnitude. We thus calculated the regression-based 95% limits of agreement as 27.7922 – 0.3332A – 1.96 × SD (of the residuals; lower limit) and 27.7922 – 0.3332A + 1.96 × SD (of the residuals; upper limit). An estimate of SD (7.6407) was calculated by the square root of total variance for all observations including the estimated between-patient variance and within-patient variance. Data were analyzed using the *nlme* package of R software version 3.6.1 (R Foundation).

## Results

Over the course of 6 months, we screened 201 consecutive patients who were scheduled for cardioversion and AF ablation procedures. Of these patients, 35 met full eligibility criteria and 22 patients were enrolled into the study (Figure 1). Demographic and clinical characteristics of the study participants are shown in Table 1. The mean age was 66 (SD 6.5) years and 85% (n=17) were male. Among the 20 participants analyzed, 3 (15%) had RVR. The majority of participants had persistent AF (14/20, 70%) and were prescribed antiarrhythmic (11/20, 55%), rate control (15/20, 75%), or anticoagulant (19/20, 95%) medications at the time of study enrollment.

Of the 280 possible HR measurements, 268 were recorded (95.7%). The first participant had 4 out of 14 recordings because the protocol was subsequently changed to capture a greater number of time points over 60 seconds of monitoring. A subsequent participant had 12 out of 14 recordings due to a failure to capture the entire 60 seconds of continuous monitoring on video. HR recordings ranged from 49 to 146 bpm from telemetry and 55 to 127 bpm from the Apple Watch.

**Figure 3:**
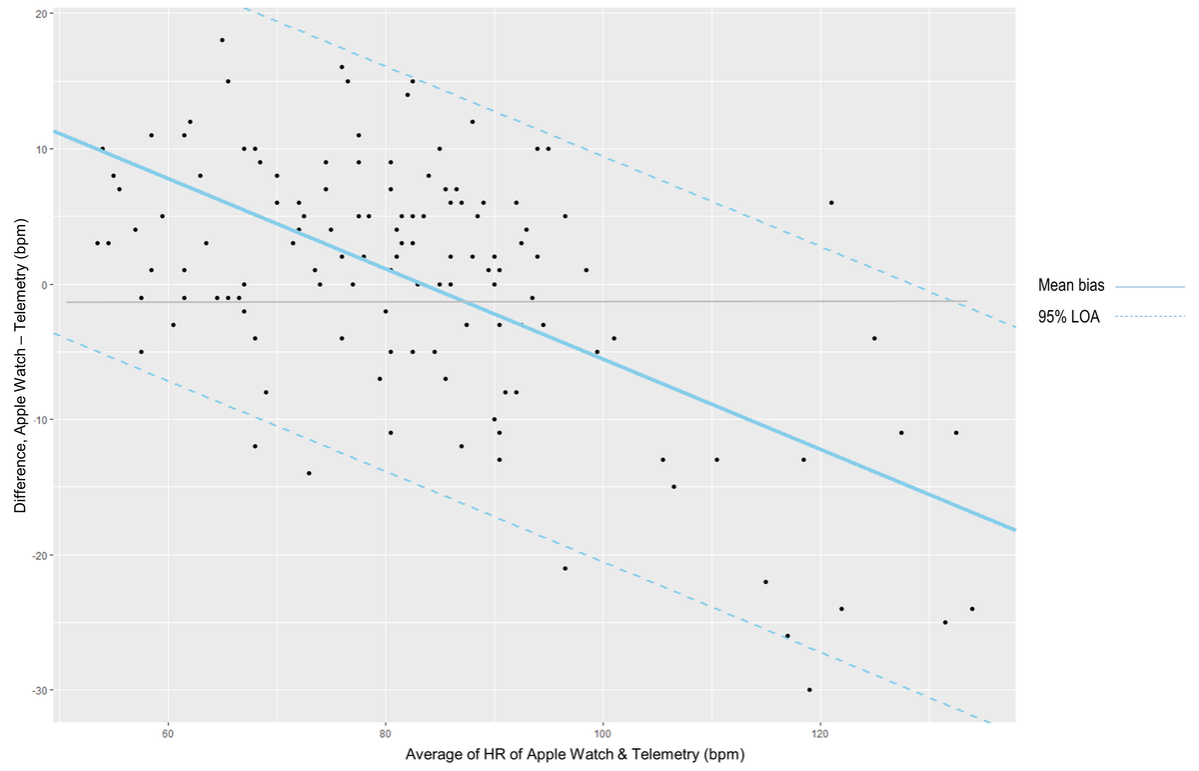
Bland–Altman plot showing 95% confidence limits with progressive increase in differences.

Figure 3 shows the standard deviation of the difference in paired measurement for each patient against the average measurement for that patient. As mentioned in the “Statistical Analysis” section, there was a suggestion that the variability of the difference increased as the magnitude of HR measurements increased. After performing the mixed effects regression model, we found that the 95% limits of agreement were calculated as 27.7922 – 0.3332A – 1.96 × 7.6407 (lower limit) and 27.7922 – 0.3332A + 1.96 × 7.6407 (upper limit), where A is the magnitude (average of 2 methods) of HR. Based on this approach, the fit was greatly improved, particularly for higher HR. The Apple Watch had 95% of differences fall within 15.0 bpm above and 15.0 bpm below telemetry measurements. Lin’s concordance correlation coefficient between the Apple Watch and telemetry is 0.88 (95% CI 0.85-0.91).

**Table 1:** Participant characteristics (N=20).

Demographic		Values
Age (years), mean (SD)		66.0 (6.5)
BMI (kg/m^2^), mean (SD)		33.2 (4.8)
**Sex, n (%)**		
	Male	17 (85)
	Female	3 (15)
**Race, n (%)**		
	White	16 (80)
	Black	4 (20)
**Atrial fibrillation, n (%)**		
	Paroxysmal	6 (30)
	Persistent	14 (70)
**CHAD-VASC^a^ score, n (%)**		
	1	8 (40)
	2	5 (25)
	3	7 (35)
**Antiarrhythmic medications, n (%)**		
	Amiodarone	8 (40)
	Dofetilide	1 (5)
	Sotalol	1 (5)
	Propenafone	1 (5)
	None	9 (45)
**Anticoagulant medications, n (%)**		
	Rivaroxaban	6 (30)
	Apixaban	8 (40)
	Dabigatran	2 (10)
	Warfarin	3 (15)
	None	1 (5)
**Rate control medications, n (%)**		
	Nadolol	1 (5)
	Metoprolol succinate	12 (60)
	Metoprolol tartrate	1 (5)
	Diltiazem	1 (5)
	None	5 (25)
**Smoking status, n (%)**		
	Current smoker	0 (0)
	Former smoker	11 (55)
	Never smoker	9 (45)
**CPAP^b^ usage, n (%)**		
	Yes	10 (50)
	Yes, but not compliant	3 (15)
	No	7 (35)

^a^CHAD-VASC: Congestive heart failure (or left ventricular systolic dysfunction), hypertension, age ≥75 years, diabetes mellitus, prior Stroke or TIA or thromboembolism, vascular disease (eg, peripheral artery disease, myocardial infarction, aortic plaque), age 65-74 years, sex category (ie, female sex).

^b^CPAP: continuous positive airway pressure.

## Discussion

### Principal Findings

This study presents a pilot effort to assess the level of agreement in HR measurements between PPG technology using the Apple Watch (1st generation) and telemetry during episodes of AF. We demonstrate that with a Lin’s concordance correlation coefficient of 0.88, the Apple Watch provided acceptable agreement with HR measurements by telemetry even during these episodes. The mean bias between the Apple Watch and telemetry measurements was 0.26 bpm, with 95% of Apple Watch HR measurements falling within 19 bpm of the telemetry measurements.

While the Lin’s concordance correlation coefficient is deemed accepted by the literature [18], we note that this interval was relatively wide, indicating there were relatively large differences in measurement. Furthermore, there appears to be an increase in variability of the differences as the magnitude of HR measurements increases, which casts doubt on the appropriateness of the constant mean bias assumption. While it is still subject to clinical judgment of how far apart HR measurements could be before 2 methods could be considered interchangeable, as Bland and Altman [19] note, the limits of agreement will be widened to some extent by the violation of the constant mean bias assumption, which thus would not lead to the acceptance of poor methods of measurement. As such, we adjusted for the average HR measurement in the mixed effects regression model to produce limits of agreement that better reflect the data [17,19].

### Limitations

Our study is not without limitations. Despite screening 201 patients over a span of 6 months, only 35 patients were eligible, due to the criteria of having objective documentation of OSA. Furthermore, as this was a pilot study and to maximize yield of HR measurements while in AF, we aimed to enroll only 20 patients, yielding 134 HR measurements for each device (268 between the Apple Watch and telemetry) for analysis. Moreover, our small sample population was skewed toward white/Caucasian males. Because enrollment occurred in the preprocedure setting among patients who have established care with an electrophysiologist, the majority of participants demonstrated good rate control, and only 15% (n=3) were in RVR. This makes it difficult to assess the accuracy of PPG technology in measuring elevated HR and detecting periods of RVR, although our data support prior work suggesting that smart watches underestimate HR in these higher ranges [20,21]. Additionally, our data were collected under the direct supervision of a team member (RS), while the participants were sedentary, ensuring adequate skin contact between the smart watch and skin to obtain HR measurements. Generalizability of our results, therefore, may be limited and further studies with a more diverse patient population and range of HR are needed, in sedentary and mobile settings. Furthermore, as our patient population was individuals with a known history of AF, our study did not demonstrate the ability to detect AF episodes, but rather the level of agreement on reported HR measurements with that of telemetry as the gold standard. This study frames the implications of our findings as an assessment of rate control rather than the actual detection of AF episodes. Regardless, we believe these data remain clinically useful for clinicians and patients aiming to evaluate adherence to treatment and titrate therapies accordingly.

### Comparison With Prior Work

Because of its clinically silent nature, AF is difficult to detect, and guideline-directed management involves anticoagulation, rate control, and rhythm control [22]. A user-friendly device that allows for passive, noninvasive, and real-time HR monitoring, even during AF episodes, would therefore have substantial clinical implications for evaluating treatment efficacy. Smart watches and other wearables may be well-positioned to provide non-obtrusive, real-time HR monitoring and AF detection over long periods, limited only by battery life, wear time, and sensor algorithms.

Although several studies have evaluated the validity of smart watch algorithms to detect AF in healthy adults without cardiovascular disease [3,6,23], while some have assessed HR accuracy in wrist-worn monitors among healthy participants or patients with cardiovascular disease [24], our work adds to the body of research by showcasing promise regarding the accuracy of HR measurement via mobile health (mHealth) technology specifically in patients who are in AF. Thus, for individuals at high risk for AF—including those diagnosed with OSA, obesity, valvular disease, or hypertension [25]—smart watches and other wearables may serve as an important clinical tool. Furthermore, for patients who are diagnosed with OSA, passive HR monitoring may be particularly beneficial for nonintrusive detection of AF. As previously noted, patients with OSA are at greater risk of AF recurrence after cardioversion, catheter ablation, and other antiarrhythmic therapies [7,10,26].

Moreover, by providing a larger cohort of data collected over a period in an ambulatory environment rather than within the restrictions of a clinic or hospital setting, smart watches have the potential to empower patients in their conversations with their health care providers regarding the efficacy of their AF therapies, including antiarrhythmic and rate control medications. This has been demonstrated in our clinical practice, where we have had patients with OSA self-identify an AF episode with RVR by a fast HR on their Apple Watch [24]. Our study may help clinicians understand the clinical utility of these ambulatory data should AF patients share the HR measurements from their Apple Watch. For patients with comorbid diagnoses of AF and OSA, the ability to passively monitor their HR with a smart watch may also promote adherence to OSA treatments including CPAP therapy and lifestyle modification, as these therapies have been shown to reduce AF recurrence and maintain sinus rhythm [26,27].

These patient–clinician conversations, informed by patient-generated data, could in turn promote adherence to guideline-directed management [28]. Current guidelines for the management of AF already address therapies including anticoagulation and rhythm control, risk factor modification (including OSA management), and remote device detection of AF through implantable devices [29]. Notably, the 2019 American Heart Association/American College of Cardiology/Heart Rhythm Society’s focused update to these guidelines remarked that “smart” or Wi-Fi-enabled devices may play a future role in the care of AF and be included in future recommendations [29]. As wearables continue to incorporate new technologies and the field of direct-to-consumer health informatics continues to evolve and address cardiovascular disease prevention and management, it is imperative that clinicians, researchers, and industry experts establish long-term collaborations to ensure that the products are accurate, safe, and beneficial without compromising clinical workflow or overwhelming the health care system.

### Conclusions

In this study, we demonstrated that during AF episodes, HR readings from a commercially available smart watch (first-generation Apple Watch) are in acceptable agreement with HR measurements by telemetry, using patients with OSA as a proxy for a high-risk population. Further studies with larger sample populations and a wider range of HR are needed to confirm these findings. As ownership of smart devices and wearables continues to grow, our work demonstrates that these devices hold promise as tools to monitor efficacy of rate control therapies for patients with AF.

## Abbreviations

AF: atrial fibrillation
CPAP: continuous positive airway pressure
HR: heart rate
ICD: implantable cardioverter-defibrillator
ILR: implantable loop recorder
OSA: obstructive sleep apnea

## References

[1] Wolf PA, Abbott RD, Kannel WB (1991). Atrial fibrillation as an independent risk factor for stroke: the Framingham Study. Stroke.

[2] Weng L, Preis SR, Hulme OL, Larson MG, Choi SH, Wang B, Trinquart L, McManus DD, Staerk L, Lin H, Lunetta KL, Ellinor PT, Benjamin EJ, Lubitz SA (2018). Genetic Predisposition, Clinical Risk Factor Burden, and Lifetime Risk of Atrial Fibrillation. Circulation.

[3] Perez MV, Mahaffey KW, Hedlin H, Rumsfeld JS, Garcia A, Ferris T, Balasubramanian V, Russo AM, Rajmane A, Cheung L, Hung G, Lee J, Kowey P, Talati N, Nag D, Gummidipundi SE, Beatty A, Hills MT, Desai S, Granger CB, Desai M, Turakhia MP, Apple Heart Study Investigators (2019). Large-Scale Assessment of a Smartwatch to Identify Atrial Fibrillation. N Engl J Med.

[4] Lin HJ, Wolf PA, Benjamin EJ, Belanger AJ, D'Agostino RB (1995). Newly diagnosed atrial fibrillation and acute stroke. The Framingham Study. Stroke.

[5] Turakhia MP, Shafrin J, Bognar K, Trocio J, Abdulsattar Y, Wiederkehr D, Goldman DP (2018). Estimated prevalence of undiagnosed atrial fibrillation in the United States. PLoS One.

[6] Mehra R, Benjamin EJ, Shahar E, Gottlieb DJ, Nawabit R, Kirchner HL, Sahadevan J, Redline S, Sleep Heart Health Study (2006). Association of nocturnal arrhythmias with sleep-disordered breathing: The Sleep Heart Health Study. Am J Respir Crit Care Med.

[7] Gottlieb DJ (2014). Sleep apnea and the risk of atrial fibrillation recurrence: structural or functional effects?. J Am Heart Assoc.

[8] Tung P, Anter E (2016). Atrial Fibrillation And Sleep Apnea: Considerations For A Dual Epidemic. J Atr Fibrillation.

[9] Gami AS, Hodge DO, Herges RM, Olson EJ, Nykodym J, Kara T, Somers VK (2007). Obstructive sleep apnea, obesity, and the risk of incident atrial fibrillation. J Am Coll Cardiol.

[10] Siontis KC, Oral H (2017). Atrial Fibrillation and Obstructive Sleep Apnea: Beyond the Pulmonary Veins. Circ Arrhythm Electrophysiol.

[11] Tajrishi FZ, Chitsazan M, Chitsazan M, Shojaei F, Gunnam V, Chi G (2019). Smartwatch for the Detection of Atrial Fibrillation. Crit Pathw Cardiol.

[12] Food and Drug Administration (FDA) (2019). 510(k) premarket notification.

[13] Food and Drug Administration (FDA) (2019). ECG App.

[14] Raja JM, Elsakr C, Roman S, Cave B, Pour-Ghaz I, Nanda A, Maturana M, Khouzam RN (2019). Apple Watch, Wearables, and Heart Rhythm: where do we stand?. Ann Transl Med.

[15] Carpenter A, Frontera A (2016). Smart-watches: a potential challenger to the implantable loop recorder?. Europace.

[16] Tison GH, Sanchez JM, Ballinger B, Singh A, Olgin JE, Pletcher MJ, Vittinghoff E, Lee ES, Fan SM, Gladstone RA, Mikell C, Sohoni N, Hsieh J, Marcus GM (2018). Passive Detection of Atrial Fibrillation Using a Commercially Available Smartwatch. JAMA Cardiol.

[17] Parker RA, Weir CJ, Rubio N, Rabinovich R, Pinnock H, Hanley J, McCloughan L, Drost EM, Mantoani LC, MacNee W, McKinstry B (2016). Application of Mixed Effects Limits of Agreement in the Presence of Multiple Sources of Variability: Exemplar from the Comparison of Several Devices to Measure Respiratory Rate in COPD Patients. PLoS One.

[18] Gillinov S, Etiwy M, Wang R, Blackburn G, Phelan D, Gillinov AM, Houghtaling P, Javadikasgari H, Desai MY (2017). Variable Accuracy of Wearable Heart Rate Monitors during Aerobic Exercise. Med Sci Sports Exerc.

[19] Bland JM, Altman DG (1999). Measuring agreement in method comparison studies. Stat Methods Med Res.

[20] Al-Kaisey AM, Koshy AN, Ha FJ, Spencer R, Toner L, Sajeev JK, Teh AW, Farouque O, Lim HS (2020). Accuracy of wrist-worn heart rate monitors for rate control assessment in atrial fibrillation. Int J Cardiol.

[21] Koshy AN, Sajeev JK, Nerlekar N, Brown AJ, Rajakariar K, Zureik M, Wong MC, Roberts L, Street M, Cooke J, Teh AW (2018). Smart watches for heart rate assessment in atrial arrhythmias. Int J Cardiol.

[22] January CT, Wann LS, Alpert JS, Calkins H, Cigarroa JE, Cleveland JC, Conti JB, Ellinor PT, Ezekowitz MD, Field ME, Murray KT, Sacco RL, Stevenson WG, Tchou PJ, Tracy CM, Yancy CW, ACC/AHA Task Force Members (2014). 2014 AHA/ACC/HRS guideline for the management of patients with atrial fibrillation: executive summary: a report of the American College of Cardiology/American Heart Association Task Force on practice guidelines and the Heart Rhythm Society. Circulation.

[23] Dörr M, Nohturfft V, Brasier N, Bosshard E, Djurdjevic A, Gross S, Raichle CJ, Rhinisperger M, Stöckli R, Eckstein J (2019). The WATCH AF Trial: SmartWATCHes for Detection of Atrial Fibrillation. JACC Clin Electrophysiol.

[24] Falter M, Budts W, Goetschalckx K, Cornelissen V, Buys R (2019). Accuracy of Apple Watch Measurements for Heart Rate and Energy Expenditure in Patients With Cardiovascular Disease: Cross-Sectional Study. JMIR Mhealth Uhealth.

[25] Genta PR, Drager LF, Lorenzi Filho G (2017). Screening for Obstructive Sleep Apnea in Patients with Atrial Fibrillation. Sleep Med Clin.

[26] Linz D, McEvoy RD, Cowie MR, Somers VK, Nattel S, Lévy P, Kalman JM, Sanders P (2018). Associations of Obstructive Sleep Apnea With Atrial Fibrillation and Continuous Positive Airway Pressure Treatment: A Review. JAMA Cardiol.

[27] Marulanda-Londoño E, Chaturvedi S (2017). The Interplay between Obstructive Sleep Apnea and Atrial Fibrillation. Front Neurol.

[28] Ying RVS, Sharma G, Martin SS (2018). Tachycardiomyopathy detection: Moving beyond arrhythmia detection with kardia band. Cardiology.

[29] January CT, Wann LS, Calkins H, Chen LY, Cigarroa JE, Cleveland JC, Ellinor PT, Ezekowitz MD, Field ME, Furie KL, Heidenreich PA, Murray KT, Shea JB, Tracy CM, Yancy CW (2019). 2019 AHA/ACC/HRS Focused Update of the 2014 AHA/ACC/HRS Guideline for the Management of Patients With Atrial Fibrillation: A Report of the American College of Cardiology/American Heart Association Task Force on Clinical Practice Guidelines and the Heart Rhythm Society. J Am Coll Cardiol.

